# Time use choices and healthy body weight: A multivariate analysis of data from the American Time use Survey

**DOI:** 10.1186/1479-5868-8-84

**Published:** 2011-08-02

**Authors:** Cathleen D Zick, Robert B Stevens, W Keith Bryant

**Affiliations:** 1 Department of Family and Consumer Studies, University of Utah, Salt Lake City, Utah, USA; 2 Department of Policy Analysis and Management, Cornell University, Ithaca, New York, USA

**Keywords:** Body mass index, time use, time spent eating, physical (in)activity time, wage rates, and grocery prices

## Abstract

**Background:**

We examine the relationship between time use choices and healthy body weight as measured by survey respondents' body mass index (BMI). Using data from the 2006 and 2007 American Time Use Surveys, we expand upon earlier research by including more detailed measures of time spent eating as well as measures of physical activity time and sedentary time. We also estimate three alternative models that relate time use to BMI.

**Results:**

Our results suggest that time use and BMI are simultaneously determined. The preferred empirical model reveals evidence of an inverse relationship between time spent eating and BMI for women and men. In contrast, time spent drinking beverages while simultaneously doing other things and time spent watching television/videos are positively linked to BMI. For women only, time spent in food preparation and clean-up is inversely related to BMI while for men only, time spent sleeping is inversely related to BMI. Models that include grocery prices, opportunity costs of time, and nonwage income reveal that as these economic variables increase, BMI declines.

**Conclusions:**

In this large, nationally representative data set, our analyses that correct for time use endogeneity reveal that the Americans' time use decisions have implications for their BMI. The analyses suggest that both eating time and context (i.e., while doing other tasks simultaneously) matters as does time spent in food preparation, and time spent in sedentary activities. Reduced form models suggest that shifts in grocery prices, opportunity costs of time, and nonwage income may be contributing to alterations in time use patterns and food choices that have implications for BMI.

## Background

The upward trend in the fraction of American adults who are overweight or obese is one of the foremost public health concerns in the United States today.^a ^The National Center for Health Statistics reports that over the past 45 years the prevalence of adult overweight (including obesity) has grown from 44.8% to 66.9% [1].^b ^Overweight and obesity are known risk factors for a number of life-threatening health conditions including coronary heart disease, stroke, hypertension, and type 2 diabetes. As a consequence, the increasing prevalence of Americans' weight problems portends a future where the billions of dollars we currently spend on overweight and obesity-related health care [2] will continue to grow and life expectancy may actually begin to decline [3].

In an effort to identify the correlates of Americans' growing overweight/obesity risk, few studies have examined the relationship between time use and BMI. Those studies that do investigate the role that time use may play generally fall into two categories. The first category includes studies where the focus is on time spent in physical activity and/or inactivity as it relates to BMI while the second category includes studies where the focus is on time spent eating and BMI.

Cross-sectional studies of physical activity time and BMI conclude that higher levels of physical activity are associated with lower BMI [4-6]. Other researchers have focused exclusively on television-viewing time or sleep time and BMI as each of these activities account for significant fractions of Americans' physically inactive time [7]. Studies focused on television/video viewing find that television time is positively related to BMI [8-10]. Those that have examined the relationship between sleep time and BMI find an inverse relationship between sleep time and BMI in the cross-section but not longitudinally [11-13].

Several studies have examined the relationship between sedentary behavior, physical activity, and BMI. One study finds a positive relationship between television viewing time and abdominal obesity risk even after controlling for leisure-related physical activity [14]. Using data from the American Time Use Survey (ATUS), another study finds that individuals who spend less than 60 minutes per day watching television/videos and who spend more than 60 minutes per day in moderate-to-vigorous leisure time physical activity have significantly lower BMIs, than otherwise comparable respondents who report spending fewer than 60 minutes watching television/videos and spending less than 60 minutes in moderate-to-vigorous physical activity [15]. Research that makes use of data from the National Health and Nutrition Examination Survey (NHANES) finds that physical activity and inactivity (measured by steps per day and time) vary significantly across normal weight, overweight, and obese individuals [16]. Finally, data from a cross-sectional Australian study reveal significant interaction effects of leisure-time sedentary and physical activities as they relate to overweight/obesity risk [17].

Fewer studies assess the relationship between time spent eating and BMI. Bertrand and Schanzenbach [18] surveyed adult women who completed a recall time diary, a dietary time diary, and reported their height and weight. Their study focuses on describing the eating context for normal and overweight women. They report that among overweight women, more calories are consumed while doing chores, socializing, relaxing, watching television, caring for others, and shopping [18].^c ^While their low cooperation rate (17 percent) and the focus only on women limits the generalizability of their study's findings, the results are nonetheless suggestive that secondary eating (i.e., eating when something else, such as television viewing, is the primary focus of an individual's time) may be linked to an increase in BMI. This contention is also supported by nutrition studies that have found that people tend to consume more calories when they are simultaneously engaged in other activities [19-24].

Hamermesh [25] uses ATUS data to explore the relationship between the price of time, time spent in primary eating and secondary eating spells (i.e., what he calls "grazing" time), the number of spells, and BMI. Using only the observations from employed individuals who report their usual weekly earnings and their usual weekly hours worked, he finds a significant inverse relationship between primary eating time and BMI. However, when number of primary eating spells is also included, the average duration of primary eating is no longer statistically significant. In addition, both average secondary spell duration and number of spells of secondary eating are generally insignificant [25].

In the research that follows, we build on these earlier studies to present a more complete picture of how time use choices may be affecting Americans' BMI. Our research builds on past investigations in several ways. First, we investigate the relationship between BMI and a range of time use categories that have typically only been examined in isolation. Specifically, we focus on physical activity time, television/video viewing time, sleep time, primary eating time, secondary eating time, and food preparation time. Second, we estimate two alternative models that allow for simultaneity in the choices individuals make about time use and BMI - something that has not been previously done. Third, we do not place any gender or employment restrictions on the sample respondents thus enhancing the external validity of our findings.

## Methods

### The 2006 and 2007 American Time Use Surveys

Data for the current investigation come from the 2006 and 2007 public-use files of American Time Use Surveys (ATUS) and have the advantage of providing valid, reliable measures of time spent in both energy intake and energy expenditure related activities over one 24-hour period [26,27]. The extraordinary level of detail in the ATUS allows us to separate time spent eating into time spent eating where eating is the respondent's primary focus and secondary eating time (i.e., time when the respondent's primary activity was something other than eating, but when eating was still taking place).

ATUS respondents are drawn from households that had completed their final interview for the Current Population Survey in the preceding 2-5 months. Each respondent is randomly selected from among each household's members, age 15 and older. Half complete a diary for a weekday and half complete a diary for a weekend day.

Information from the ATUS interviews is linked to information from the 2006 and 2007 Eating and Health module interviews [28,29] so that we also have data on the respondent's height and weight. BMI is calculated from self-reported weight in kilograms divided by self-reported height in meters squared. It should be noted that although self-reported BMI has been commonly used in past studies [30-34], some have found that it results in a modest under-estimation of overweight and/or obesity rates [35-37] while others have found it to be a valid and reliable way to measure BMI for nonelderly adults [38].

We restrict our ATUS sample to those respondents who are between the ages of 25 and 64, inclusive. Younger respondents are excluded so as to avoid the inclusion of individuals whose eating habits may be dictated by their parents. Respondents over age 64 are excluded because these individuals are more likely to have health conditions that may affect some aspects of their time use. We also restrict our sample to those respondents whose BMI ranges from 16.0 to 60.0, inclusive. These BMI restrictions lead to the elimination of 5 male respondents (1 with BMI > 60.0 and 4 with BMIs < 16.0) and 17 female respondents (5 with BMIs >60.0 and 12 with BMIs < 16.0). In addition, we eliminate 12 respondents who report spending more than 15 hours being physically active, 18 respondents who report spending more than 20 hours sleeping and 4 respondents who report spending more than 20 hours watching television. These restrictions are made to reduce the potential influence of leverage points and outliers. Finally, we exclude women who are pregnant as their reported BMIs are likely not reflective of their usual BMIs. These sample restrictions result in a sample of 8,856 women and 7,586 men in our study.

We focus on seven time-use categories that are potentially related to energy balance. The first category measures the amount of primary time the respondent spends eating and drinking (i.e., time where eating and drinking has her/his primary attention).^d ^Secondary eating time is captured by the amount of time the respondent reports eating as a secondary activity (i.e., time where something else has her/his primary attention). Secondary time spent drinking anything other than plain water is measured separately. Other food related activities are measured by the time spent in food preparation and clean-up excluding related travel time.

Physical activity cannot be adequately measured by simply summing the time respondents report spending in exercise and sports as we would end up omitting things like bicycling to work, chasing after a toddler, and doing physically demanding household chores. Thus, rather than use only time spent in the ATUS sports and exercise categories, we sum time spent in all activities in the ATUS activity lexicon that generate metabolic equivalents (METs) of 3.3 or more. We select these activities based on the work done by Tudor-Locke et al. [39] who have linked the ATUS time use lexicon to the Compendium of Physical Activities. We choose a threshold of 3.3 METs because this captures activities such as exterior house cleaning, lawn and garden work, caring for and helping household children, playing sports with household children, active transportation time (i.e., walking or biking), as well as most forms of sports, exercise, and recreation. It excludes such routine household activities such as interior housekeeping and playing with children in non-sports.^e ^The compendium also identifies time spent in certain occupations (i.e., building and grounds cleaning and maintenance, farming, construction and extraction) as generating a minimum of 3.3 METs. To control for occupational physical activity requirements, we include a dummy variable in the male equation that takes on a value of "1" if the respondent works in one of these occupational categories. Only a handful of female respondents report working in these fields and thus we exclude this dummy from the female regressions. We sum only spells of 10 minutes or more of physical activity time because prior work has established 10 minutes as the minimum duration necessary to impact an individual's energy balance [40].

Finally, we use two measures of inactivity: television/video viewing time and time spent sleeping. These two measures have been associated with BMI and/or obesity risk in previous studies that have related single categories of time use to BMI [8,9,11-14].

### Analysis Approach

To examine the relationship between time use and BMI, ideally one would have longitudinal data on time use in various activities. Unfortunately, longitudinal time diary data do not exist. While some surveys do gather information on typical time use, methodological research has shown such questions provide less valid and reliable measures when compared to diary data [26,27,41].

Conceptually, cross-sectional time diary data of the type available in the ATUS have two disadvantages. First, time spent in various activities on any given day may deviate from an individual's usual time use patterns. As such, there is measurement error in the independent time use variables that likely bias the coefficient estimates toward zero [42]. Second, any observed association between time use and BMI obtained using cross-sectional data may reflect reverse causality. For example, having a high BMI may lead one to spend less time being physically active. To address both data shortcomings, we adopt a model of time use where BMI and time use are simultaneously determined.

In our model, BMI is a function of time use, biological traits (e.g., age, gender, race/ethnicity, health status) and socio-demographic characteristics (e.g., marital status, number of children, employment status, and education). Decisions about how much time to spend in various activities is a function of household roles (e.g., self-identification as the primary meal preparer, self-identification as the primary grocery shopper), structural factors (e.g., number of children in the home, marital status, employment status, gender, race/ethnicity, age, weekend or weekday diary, season of the year, rural residence, region of residence), prices (e.g., the respondent's wage rate, grocery prices), and income.

Data on wage rates in the ATUS are limited to those individuals who report both hours of work and earnings. To avoid the possibility of selection bias that could be introduced by excluding those who are not employed, we elect to use predicted hourly opportunity costs of time generated from wage regressions estimated using the corresponding years of the March Supplement to the Current Population Survey (CPS). We use individuals age 25-64 in the March Supplement to estimate wage equations that correct for sample selection bias using the techniques developed by James Heckman [43]. Equations are estimated separately for women and men using the appropriate CPS weights. Coefficients from these equations are used to generate predicted hourly opportunity cost of time for each individual in our ATUS sample. A random error is added to each predicted wage based on a mean of zero and a variance that is equal to the variance of the estimating equation.^f ^Estimates of offered wage rates provide approximate opportunity cost estimates of the value of time for employed individuals and lower-bound estimates of the value of time for non-employed individuals [43].

The ATUS contains a categorical measure of annual household income. The categorical nature of this variable coupled with item-specific non-response made it less than ideal to use on our analyses. Consequently, we again turn to the March Supplement to the CPS. For individuals age 25-64, we estimate a regression using the appropriate CPS weights where total, annual nonwage income for the household is the dependent variable. Coefficients from this equation are then used to generate predicted nonwage income values for our sample of respondents in the ATUS. A random error is added to each predicted nonwage income value based on a mean of zero and a variance that is equal to the variance of the estimating equation.^g^

Grocery price information comes from the Council for Community and Economic Research's (C2ER) state-based cost of living index for 2006 and 2007. C2ER provides expenditure weighted, quarterly metropolitan and micropolitan price information [44].^h ^The only detailed geographic information contained in the ATUS is the respondent's state of residence and residential urbanicity. Thus, our linkage of grocery price information is done based on information about the respondent's state of residence, urban/rural status, and the quarter in which the respondent was interviewed. In those rare cases where the respondent was located in a micro area within a state that had no micro grocery price index, we use the state-wide average. Initially, we also included an index measuring non-grocery prices but this was dropped from our analyses once it was determined that the simple correlation between the grocery price index and the non-grocery price index was .89.

We estimate three different sets of equations separately for men and women. In the first formulation, we estimate a model where our time use measures are treated as predetermined variables that affect BMI. We then estimate an instrumental variables model that recognizes that the time use and BMI causality may run in both directions when one is analyzing cross-sectional data of the sort used here. In the final formulation, we estimate reduced form models of BMI. In this formulation, BMI is estimated as a function of the biological and socio-demographic variables and the strictly exogenous factors that are posited to affect time use [45]. Essentially, these latter two estimation approaches both incorporate the hypothesis that time use and BMI are simultaneously determined.

Key to identifying the preferred model is undertaking tests for endogeneity and then, if endogeneity is confirmed, identifying "instruments" that are correlated to time use but unrelated to the error term in the BMI equation [45]. We test for endogeneity by estimating the Durbin-Wu-Hausman F-statistic [46]. Strength of the instruments is assessed by calculating a variation on the squared partial correlation between the instruments excluded from the second stage and the endogenous regressors [47]. Independence of the instruments from the error term in the BMI equation is assessed by calculating Hansen's J statistic [46].

The instrumental variables used to identify the system in our application are self-identification as the primary meal preparer, self-identification as the primary grocery shopper, whether the diary day was a weekend, whether the diary day was in the summer, whether the diary day came from 2007, the grocery price index, the hourly opportunity cost of time, and the household's annual nonwage income. The instrumental variables approach involves first estimating the time use equations and using the coefficients from these equations to generate predicted time use values for all respondents in the sample. These predicted values are then included as regressors in the BMI equations. If all of the necessary conditions are met, the estimated coefficients using this approach are purged of possible reverse causation. This approach has the added advantage of also addressing the typical time use measurement issue since the predicted values may be thought of as approximating usual time spent in the various activities.

Separate equations are estimated for women and men to allow for the possibility that there are biological factors related to gender that interact with time use and are associated with BMI. All analyses are weighted using the appropriate ATUS weights. The ATUS weights compensate for the survey's oversampling of certain demographic groups, the oversampling of weekend day diaries, and differential response rates across demographic groups [48]. Estimation is done using Stata 11.0 and SAS 9.2.

## Results

### Sample Characteristics

Descriptive statistics for our samples of men and women appear in Table 1. The typical male in our sample is about 44 years old, married, and has one minor child in the home. He is often the primary grocery shopper (most often when he is not married), but not the primary meal preparer in his household. He has some college education and is currently employed. His hourly opportunity cost of time is almost $21/hr and he lives in a household that has approximately $1,669 in nonwage income per year. The typical female respondent in our sample is very similar. She is also 44 years old, married, and has one minor child in the home. She is most often both the primary grocery shopper and the primary meal preparer. She has some college education and lives in a household that has approximately $1,604 in nonwage income per year. The hourly opportunity cost of her time is lower at $16.84/hr, about 80% of her male counterpart's, and she is also employed outside of the home.

**Table 1:** Weighted Descriptive Statistics

		Males (N = 7,586)		Females (N = 8,856)	
**Variable**	**Definition**	**Mean/Proportion**	**Standard Deviation**	**Mean/Proportion**	**Standard Deviation**

**BMI**	weight in kilograms divided by the square of height in meters	28.31	5.13	27.33	6.25

**Overweight/Obese**	1 = BMI > 25.0	.75		.57	

	0 = BMI ≤25.0				

**Age**	Age in years	43.69	10.92	44.19	10.90

**Married/Cohabitating**	1 = married or cohabitating	.71		.69	

	0 = not married or cohabitating				

**Number of Kids < Age 6**	number	.28	.63	.28	.61

**Number of Kids Age 6-17**	number	.58	.94	.65	.98

**Education**	Years of formal schooling	13.66	2.67	13.73	2.51

**Occupation with METs > 3.3**	1 = working in building/grounds maintenance, farming, fishing, forestry, construction, or extraction,	.10		---	

	0 = otherwise				

**Employed**	1 = currently employed	.83		.70	

	0 = not currently employed				

**Poor Health**	1 = respondent says health is currently fair or poor	.15		.15	

	0 = otherwise				

**Primary Meal Preparer^a^**	1 = primary meal preparer in the household	.39		.83	

	0 = otherwise				

**Primary Grocery Shopper^a^**	1 = primary grocery shopper in the household	.52		.90	

	0 = otherwise				

**Weekend**	1 = time diary comes from a weekend day	.29		.29	

	0 = time diary comes from a weekday				

**Summer**	1 = time diary comes from a summer month	.25		.25	

	0 = otherwise				

**Black^b^**	1 = Black, non-Hispanic	.11		.13	

	0 = otherwise				

**Hispanic^b^**	1 = Hispanic	.13		.12	

	0 = otherwise				

**Other^b^**	1 = race/ethnicity something other than Black non-Hispanic, Hispanic, or White non-Hispanic	.05		.06	

	0 = otherwise				

**ATUS07**	1 = respondent in the 2007 ATUS	.50		.50	

	0 = respondent in the 2006 ATUS				

**Grocery Price Index**	ACCRA state-level grocery price index: 2006	103.21	10.51	102.99	10.60

**Hourly Opportunity Cost of Time**	$/hour	20.57	7.74	16.84	5.27

**Ln(Non-Wage Income)**	Ln($ per year from all nonwage sources in the household)	7.42	0.57	7.38	0.56

^**a**^Note that the fraction of women and men who identify themselves as the primary meal preparer (grocery shopper) will sum to more than 100 percent because approximately 30 percent of men and women in the sample are single non-cohabitating individuals.

^**b**^The omitted category in this sequence of dummy variables are those respondents who are White and Non-Hispanic.

Table 1 also reveals that the typical man and woman in our sample are overweight (defined by a BMI that is greater than 25.0 and less than 30.0). Indeed, fully 75 percent of the males in our sample are overweight or obese while the corresponding figure for the females is lower at 57 percent. As a point of comparison, analysis of clinical data from the National Health and Nutrition Examination Survey (NHANES) show that in 2003-06, 72.6 percent of males age 20-74 and 61.2 percent of females age 20-74 were overweight or obese [1]. While the years and our sample age ranges are not entirely comparable to those in the NHANES study (i.e., our sample age restriction is 25-64), the figures nonetheless suggest that, on average, the self-reported height and weight in the ATUS do a reasonable job of classifying adults' BMIs. In a more extensive comparison of ATUS BMI measures to NHANES BMI measures, Hamermesh [23] reaches the same conclusion for men but notes a modest downward bias in BMI reporting for women in the ATUS relative to NHANES.

The descriptive information on the time-use measures appears in Table 2. It shows that women and men, respectively, spend an average of a little more than an hour a day in eating where that is the main focus of their attention. They also spend more than 20 minutes per day on average engaged in eating as a secondary activity.^i ^Secondary time spent drinking is much higher with the average time being 57 minutes for men and almost 69 minutes for women. Time spent in food preparation and clean-up is substantially greater for women than men (about 2.6 times more). Physically active time averages about 68 minutes a day for men and 35 minutes a day for women. Sleep time averages a little more than 8 hours for both men and women. Finally, the typical woman and man both spend considerable time watching television/videos, with men averaging 2.67 hours per day and women averaging 2.13 hours per viewing television/videos.

**Table 2:** Descriptive Statistics for the Time Use Measures

		Males				Females			
**Time Use Variable**	**Definition**	**Overall Mean**	**Standard Deviation**	**Percent Non-Zero**	**Non-Zero Mean**	**OverallMean**	**Standard Deviation**	**Percent Non-zero**	**Non-Zero Mean**

**Primary Eating Time**	Total minutes over 24 hr (10 min. increments)	6.83	4.91	.96	7.11	6.44	4.72	.96	6.76

**Secondary Eating Time**	Total minutes over 24 hr (10 min. increments)	2.15	8.51	.52	4.28	2.26	8.81	.59	3.85

**Secondary Drinking Time**	Total minutes over 24 hr (10 min. increments)	5.74	16.82	.36	16.20	6.89	18.62	.41	16.56

**Food Preparation Time**	Total minutes over 24 hr (10 min. increments)	1.86	3.87	.43	4.60	4.79	6.08	.71	6.83

**Physical Activity Time**	Total Minutes over 24 hr (10 min. increments)	6.77	16.79	.41	22.26	3.54	9.24	.32	11.27

**Sleep Time**	Total minutes over 24 hr (10 min. increments)	49.38	12.88	.99	49.44	49.98	12.46	.99	50.01

**Television/Video Viewing Time**	Total minutes over 24 hr (10 min. increments)	16.04	16.01	.81	19.70	12.81	13.50	.77	16.71

Also presented in Table 2 are the fractions of respondents who spend any time in each of the seven activities on the diary day. Note that virtually all respondents report that they spend some time engaged in eating as a primary activity and sleep. However, for most other activities, there are substantial numbers who report no time being spent in a particular time-use category. The censored distribution of time use leads us to use a tobit routine to estimate the first stage in our instrumental variables analyses.

### Multivariate Results

Table 3 shows the parameter estimates for all three models for both women and men. The ordinary least squares (OLS) model suggests that all seven time use categories are linked to BMI while the instrumental variables model indicates that only a subset of the time use categories relate to BMI. Which model is to be preferred? The answer to that question hinges on three things: (1) an evaluation of whether endogeneity exists, (2) the strength of the instruments used to address any observed endogeneity, and (3) the independence of the instruments from the error process.

**Table 3:** Weighted BMI Parameter Estimates (t ratios in parentheses)

	Males			Females		
**Independent Variables**	**OLS Model**	**Instrumental Variables Model**	**Reduced Form Model^a^**	**OLS Model**	**Instrumental Variables Model**	**Reduced Form Model^a^**

**Intercept**	30.23 (54.86)**	33.22 (16.84)**	38.30 (21.38)**	29.98 (46.18)**	30.00 (11.74)**	35.40 (18.02)**

**Primary Eating Time^a^**	-.03 (-2.10)**	-0.74 (-2.42)**		-.03 (-2.30)**	-0.66 (-2.46)**	

**Secondary Eating Time^a^**	-.02 (-3.56)**	-0.96 (-3.06)**		-.03 (-4.40)**	-0.37 (-2.28)*	

**Secondary Drinking Time^a^**	.01 (2.49)**	2.14 (1.87)*		.01 (2.32)**	0.36 (1.81)*	

**Food Preparation Time^a^**	-.05 (-3.07)**	0.04 (.35)		-.03 (-2.57)**	-0.17 (-2.75)**	

**Physically Active Time^a^**	-.01 (-2.11)**	0.02 (.58)		-.02 (-3.88)**	0.37 (.49)	

**Sleep Time^a^**	-.02 (-4.36)**	-0.14 (-2.48)**		-.00 (0.40)	-0.04 (-.47)	

**Television/Video Time^a^**	.01 (3.50)**	0.18 (4.23)**		.03 (5.30)**	0.19 (2.05)**	

**Age**	.01 (1.57)	0.00 (.10)	.09 (5.39)**	.03 (5.10)**	0.05 (2.80)**	.07 (4.36)**

**Black**	.18 (.92)	-1.56 (-2.38)**	.01 (.06)	2.42 (12.17)**	1.24 (3.45)*	2.35 (11.57)**

**Hispanic**	.09 (.51)	0.37 (1.80)*	-.18 (-.94)	.76 (3.65)**	1.52 (5.77)**	.76 (3.52)**

**Other**	-1.04 (-3.80)**	-0.67 (-2.19)**	-.72 (-2.57)**	-.64 (-2.30)**	0.69 (1.84)*	-.51 (-1.77)*

**Married/Cohabitating**	.69 (4.87)**	1.14 (4.54)**	.22 (1.18)	-.45 (-3.10)**	0.27 (1.04)	-.74 (-4.57)**

**Education**	-.17 (-6.89)**	0.18 (2.06)**	-.06 (-1.21)	-.34 (-12.22)**	-0.09 (-1.18)	-.23 (-4.54)**

**Employed**	.47 (2.72)**	1.25 (4.72)**	.44 (2.62)**	.23 (1.52)	0.35 (.64)	.16 (1.07)

**Poor Health**	2.21 (12.73)**	1.39 (4.83)**	2.27 (13.10)**	3.04 (16.03)**	2.31 (8.60)**	3.15 (16.61)**

**Occupation with METs > 3.3**	-.54 (-3.08)**	-0.43 (-.69)	-.75 (-4.88)**	---	---	---

**Number of Kids < Age 6**	-.07 (-.72)	0.25 (1.92)*	-.24 (-2.24)**	-.04 (-.36)	0.34 (1.87)*	-.08 (-.70)

**Number of Kids Age 6-17**	.04 (.69)	0.04 (.53)	.07 (1.10)	.00 (.05)	0.15 (1.00)	.02 (.33)

**Weekend**			.06 (.52)			-.04 (-.27)

**Primary Meal Preparer**			-.07 (-.46)			-.71 (-3.73)**

**Primary Grocery Shopper**			.19 (1.35)			.22 (.94)

**Summer**			.24 (1.82)*			.10 (.69)

**ATUS07**			.05 (.45)			-.00 (-.00)

**Grocery Price Index**			-.03 (-4.69)**			-.03 (-4.50)**

**Hourly Opportunity Cost of Time**			-.06 (-2.89)**			-.07 (-2.75)**

**Ln(Non-Wage Income)**			-1.34 (-4.68)**			-.55 (-1.75)**

**Adjusted R^2^**	.05	.05	.05	.11	.11	.11

**F-Statistic**	23.47**	21.92**	22.19**	67.53**	65.34**	62.22**

*p < .10, **p < .05

^a ^Time use is measured in 10 minute increments.

To test for endogeneity, we first estimate the reduced form equations for time use. The residuals from these equations are then included as additional regressors in the structural equations. The Durbin-Wu-Hausman F-statistic assesses if the residuals are statistically significant which would imply that time use and BMI are endogenous [46]. Our set of seven time use categories have an associated F-statistic of 4.92 (p < .01) for males and 5.01 (p < .01) for females. Thus, we are confident that endogeneity exists.

Shea's partial R^2 ^statistic can be used to assess the strength of a set of instruments adjusting for their inter-correlations when estimating an OLS regression. However, in our case the censored nature of the dependent variables leads us to estimate the time use equations using tobit rather than OLS. Consequently, we assess instrument strength by estimating the χ^2 ^associated with the instruments excluded from the second stage estimation and each endogenous regressor. This approach is parallel to an OLS approach suggested by Bound, Jaeger, and Baker [47]. The calculated χ^2 ^for males ranges from a low of 72 in the case of secondary eating time to a high of 722 for television/video viewing time. For females, the range is 136 (secondary drinking time) to 496 (sleep time). All are far above the critical χ^2 ^of 21.67, suggesting that our instruments are strong.

Independence of the instruments is assessed by Hansen's J statistic which has a χ^2 ^distribution with degrees of freedom equal to the number of over-identifying restrictions [46]. A statistically significant value suggests that the instruments used in the first stage are *not *independent of the second stage error term. In our model, Hansen's J is 3.03 (p = .22) for women and 2.33 (p = .31) for men, indicating the instruments are not associated with the error term in either instance.

Taken altogether, the above statistical tests indicate that there is endogeneity between time use and BMI and that the instruments used in our estimation meet the criteria necessary to rely on the instrumental variables approach. Thus, we highlight the results for the second stage instrumental variables model along with the alternative reduced form estimates. Parameter estimates of the first stage estimation appear in Appendix Tables 4 and 5 for the reader's reference.

**Table 4:** First Stage Parameter Estimates from the Tobit Equations: Males (t ratios in parentheses)^a^

Independent Variables	Primary Eating Time	Secondary Eating Time	Secondary Drinking Time	Food Preparation Time	Physical Activity Time	Sleep Time	Television/video Time
**Intercept**	-1.59 (-.90)	-10.16 (-1.93)*	-30.00 (-2.04)**	-9.72 (-3.29)**	-19.45 (-1.66)*	49.95 (11.33)**	31.31 (4.97)**

**Age**	0.01 (.31)	-0.06 (-1.24)	-0.16 (-1.20)	0.07 (2.68)**	.22 (2.06)**	-0.18 (-4.51)**	0.09 (1.58)*

**Married/Cohabitating**	0.70 (3.85)**	-0.29 (-.54)	-0.73 (-.49)	0.89 (2.92)**	2.31 (1.89)*	-0.75 (-1.67)*	-1.21 (1.88)*

**Education**	0.23 (4.97)**	0.55 (3.94)**	1.68 (4.27)**	0.04 (.55)	-.48 (-1.54)	-0.62 (-5.42)**	-.97 (-5.90)**

**Black**	-2.04 (-9.80)**	0.56 (.92)	-9.13 (-5.23)**	0.34 (.97)	-4.67 (-3.26)**	0.72 (1.41)	1.68 (2.29)**

**Hispanic**	0.09 (.48)	-4.12 (-6.77)**	-16.61 (-9.40)**	-0.58 (-1.74)*	3.49 (2.73)**	2.07 (4.26)**	-.17 (.25)

**Other**	-0.06 (-.22)	-3.49 (-4.11)**	-11.36 (-4.75)**	0.97 (2.08)**	-.56 (-.29)	1.63 (2.36)**	-0.60 (-.61)

**Occupation with METs > 3.3**	0.47 (3.04)**	-2.64 (-5.63)**	-4.80 (-3.65)**	1.09 (4.25)**	34.97 (36.12)**	.12 (.33)	-1.72 (-3.16)**

**Fair/Poor Health**	-0.56 (-3.25)**	-1.55 (-2.94)**	-2.22 (-1.51)	-0.05 (-.18)	-4.57 (-3.97)**	1.42 (3.34)**	3.56 (5.87)**

**Employed**	-0.28 (-1.66)*	0.45 (.90)	0.81 (.58)	-1.37 (-4.95)**	-6.29 (-5.74)**	-3.37 (-8.11)**	-7.69 (-12.98)**

**Grocery Price Index**	0.01 (1.80)*	0.02 (.92)	-0.10 (-2.17)**	0.01 (1.36)	-.01 (-.23)	0.02 (1.44)	-0.03 (-1.58)

**Weekend**	0.56 (4.41)**	0.78 (2.12)**	-.88 (.86)	0.49 (2.32)**	3.57 (4.33)**	6.61 (21.13)**	7.40 (16.56)**

**Primary Grocery Shopper**	0.17 (1.26)	0.51 (1.25)	-0.05 (-.04)	0.78 (3.32)**	.62 (.67)	-0.71 (-2.06)**	-1.39 (-2.83)**

**Primary Meal Preparer**	-0.27 (-1.81)*	-0.18 (-.40)	-0.40 (-.32)	4.06 (16.07)**	-.54 (-.54)	0.05 (.14)	0.51 (.94)

**Summer**	-0.10 (-.73)	-0.10 (-.25)	0.37 (.34)	-0.36 (-1.60)	5.22 (6.07)**	0.58 (1.77)*	-1.06 (-2.27)**

**ATUS07**	0.12 (1.01)	1.26 (3.63)**	4.46 (4.63)**	0.82 (4.11)**	1.64 (2.09)**	0.19 (.66)	0.65 (1.56)

**Hourly Opportunity Cost of time**	0.00 (.05)	-0.08 (-1.38)	-.24 (-1.51)	0.04 (1.18)	.21 (1.67)*	0.14 (2.91)**	-0.11 (-1.54)

**Ln (Non-Wage Income)**	0.50 (1.77)*	0.19 (.23)	-1.99 (.85)	-0.16 (-.33)	-.17 (-.09)	1.64 (2.33)**	0.56 (.55)

**Number of Kids < Age 6**	0.15 (1.42)	0.27 (.87)	-.32 (-.37)	1.14 (6.57)**	1.85 (.2.67)**	-0.23 (-.88)	-1.80 (-4.79)**

**Number of Kids Age 6-17**	-0.17 (-2.67)**	0.04 (.24)	1.02 (1.95*	0.59 (5.45)**	.22 (.52)	-0.58 (-3.61)**	-1.24 (-5.39)**

*p < .10, **p < .05

^a ^Time use is measured in 10 minute increments.

**Table 5:** First Stage Parameter Estimates from the Tobit Equations: Females (t ratios in parentheses)^a^

Independent Variables	Primary Eating Time	Secondary Eating Time	Secondary Drinking Time	Food Preparation Time	Physical Activity Time	Sleep Time	Television/video Time
**Intercept**	-2.59 (-1.61)*	15.67 (3.50)**	18.43 (1.42)	0.17 (.07)	10.95 (1.28)	58.22 (14.52)**	29.08 (5.41)**

**Age**	-0.03 (-2.33)**	0.13 (3.57)**	0.24 (2.20)**	0.11 (4.81)**	.37 (5.08)**	-0.15 (-4.34)**	0.04 (.78)

**Married/Cohabitating**	0.72 (5.41)**	-0.63 (-1.71)*	-0.98 (-.93)	2.44 (11.26)**	0.40 (.57)	-0.94 (-2.83)**	-1.15 (-2.59)**

**Education**	0.07 (1.75)*	0.44 (3.87)**	1.24 (3.72)**	-0.32 (-4.95)**	-0.04 (-.21)	-0.52 (-5.11)**	-0.76 (-5.50)**

**Black**	-1.01 (-6.09)**	-1.35 (-2.91)**	-12.84 (-9.33)**	-0.30 (-1.12)	-4.32 (-4.68)**	1.71 (4.14)**	2.24 (4.03)**

**Hispanic**	0.56 (3.06)**	-3.73 (-7.33)**	-19.02 (-12.24)**	1.96 (6.92)**	-4.79 (-4.91)**	1.54 (3.46)**	-0.70 (-1.17)

**Other**	0.78 (3.28)**	-2.24 (-3.36)**	-12.70 (-6.40)**	2.37 (6.28)**	-3.03 (-2.38)**	1.37 (2.33)**	-2.48 (-3.08)**

**Fair/Poor Health**	-0.67 (-4.29)**	-1.34 (-3.05)**	2.15 (1.70)*	0.47 (1.88)*	-3.40 (-4.03)**	0.97 (2.50)**	2.34 (4.52)**

**Employed**	-.57 (-4.89)**	-0.86 (-2.55)**	3.10 (3.15)**	-2.12 (-11.04)**	-3.30 (-5.24)**	-1.80 (-5.97)**	-5.52 (-13.70)**

**Grocery Price Index**	0.02 (3.10)**	0.010 (.35)	-0.04 (-.99)	0.01 (.80)	0.11 (3.93)**	-0.01 (-.50)	-0.02 (-1.45)

**Weekend**	0.86 (7.59)**	-0.44 (-1.39)	-1.63 (-1.78)*	0.18 (.80)	3.06 (5.18)**	5.84 (20.63)**	4.07 (10.73)**

**Primary Grocery Shopper**	-0.17 (-.90)	-0.19 (-.36)	1.31 (.86)	1.42 (4.51)**	4.50 (4.19)**	-0.81 (-1.70)*	-1.21 (-1.89)*

**Primary Meal Preparer**	-0.20 (1.26)	.37 (.86)	-3.04 (-2.49)**	2.80 (10.90)**	2.61 (3.07)**	0.01 (.02)	-0.13 (-.26)

**Summer**	0.14 (1.19)	1.37 (4.23)**	3.43 (3.65)**	-.27 (-1.42)	3.70 (6.02)**	-0.24 (-.83)	-0.62 (-1.56)

**ATUS07**	-.14 (-1.30)	2.04 (7.11)**	4.79 (5.74)**	0.18 (1.06)	-1.28 (-2.31)**	-0.50 (-1.94)*	0.18 (.52)

**Hourly Opportunity Cost of time**	0.04 (2.14)**	-0.07 (-1.33)	-.48 (-2.97)**	0.07 (2.14)**	-0.11 (-1.05)	0.15 (2.93)**	-0.16 (-2.30)**

**Ln (Non-Wage Income)**	0.95 (3.67)**	-3.84 (-5.31)**	-5.93 (-2.97)**	-.60 (-1.43)	-6.72 (-4.81)**	0.61 (.94)	0.15 (.17)

**Number of Kids < Age 6**	-0.11 (-1.10)**	-0.28 (-.55)	-0.91 (-1.16)	1.25 (1.11)	-3.04 (-5.61)**	-0.55 (-2.26)**	-1.81 (-5.53)**

**Number of Kids Age 6-17**	-.32 (-5.69)**	-0.09 (-.55)	.08 (.17)	1.11 (12.39)**	-0.65 (-2.16)**	-0.24 (-1.70)*	-1.02 (-5.41)**

*p < .10, **p < .05

^a ^Time use is measured in 10 minute increments.

It is important to note that the time use coefficients estimated in the instrumental variables formulation are always larger than their counterpart estimates in the OLS model. This is not surprising as past research has demonstrated that "small window" measurements of the type provided in a 24-hour time diary are likely biased toward zero in multivariate analyses [42]. In this context, the instrumental variables approach is also preferred as it provides estimates of the relationship between typical time use, rather than a single day's report of time use, and BMI.

For both females and males, an increase in either primary or secondary eating time is associated with a significantly lower BMI while an increase in secondary drinking time translates into a significant increase in BMI. Increases in television/video time are also associated with a statistically significant increase in BMI for both men and women. An increase in sleep time is linked to a significant decline in BMI for men but not women while more time spent in food preparation is associated with a decline in BMI for women but not men. Although time spent being physically active had a significant negative relationship to BMI in the OLS model, this relationship is not present for either women or men in the instrumental variables estimates. We attribute this null finding to the "small window" problem associated with a single 24-hour time diary as physical activity, particularly exercise and sports, may not occur on a daily basis. With the exception of secondary eating time, the signs of all the statistically significant coefficients are in keeping with our hypotheses.

The instrumental variables specification reveals several differences in socio-demographic variables by gender. Age, race/ethnicity, marital status, education, and employment effects all vary by gender. For example, an increase in age is associated with a statistically significant increase in BMI for women but not men. Conversely, married/cohabitating males have significantly higher BMI's than single males, while marriage/cohabitation has no effect on BMI for women, *ceteris paribus*. One of the few socio-demographic variables that do not vary by gender is health status. Being in fair/poor health is associated with a large increase in BMI for both women and men.

The reduced form estimates also demonstrate considerable socio-demographic differences by gender. But, they reveal striking similarities with regard to the economic variables. For both women and men, increases in grocery prices, opportunity costs of time, and nonwage income are all associated with significantly lower BMI.

## Discussion

Our analyses reveal consistent evidence that primary eating time is inversely related to BMI. Other time diary research has found that Americans' time spent in primary eating activities has declined by an average of 11 minutes per day for women and 23 minutes per day for men between 1975 and 2006 [49]. Taken together with the findings of this earlier study, the current research suggests that the rise in BMI over the past 30+ years may be associated, in part, with changes in Americans' time spent in primary eating activities. Specifically, based on our instrumental variables model, we estimate that an 11-minute decline per day in women's primary eating time may have translated into a .73 increase in BMI for women. Likewise, a 23 minute per day decline in primary eating time over this historical period would translate into 1.70 increase in BMI for men.

While time spent in primary eating activities has declined, trend analyses of time diary data show that secondary eating and drinking time has risen from an average of 20 minutes per day for women in 1975 to 80 minutes per day in 2006-07. Similarly, men's secondary eating and drinking time has risen from an average of 25 minutes per day to 70 minutes per day over that same historical period [49]. Surprisingly, in the instrumental variables model, secondary eating time is associated with a significantly lower BMI for both men (p < .05) and women (p < .10). But, secondary drinking time is associated with higher men's and women's BMIs (p < .10). Our descriptive statistics reveal that secondary drinking time makes up approximately three-quarters of all time spent in secondary eating and drinking activities. Past studies have found a positive relationship between secondary eating and drinking time and BMI for women [18,19] while others [25] find little evidence of secondary eating/drinking effects on BMI. Ours is the first to parse out secondary eating and drinking time. As such, it sheds some light on the mixed findings in the literature, pointing the finger to increases in secondary drinking time (rather than secondary eating time) as a possible contributing factor to rising BMIs.

The social-psychological literature would suggest that less monitoring of caloric intake should occur when eating and drinking occur a secondary activities and thus time spent in these activities should be associated with higher BMI [20-23]. We find this is true with respect to secondary drinking but not secondary eating. We do not have a ready explanation for the inverse relationship between secondary eating time and BMI. Given the limited and very mixed evidence regarding any possible linkage between time spent in secondary eating activities and BMI, further research on this point is sorely needed.

Findings regarding the role that food preparation time plays in BMI are intriguing. For women, the more time spent in food preparation and clean-up, the lower their BMIs. Presumably, more time spent in food preparation and clean-up is associated with using more primary foods and fewer prepared foods when cooking. It may also be associated with smaller serving sizes relative to those found in prepared meals. Since 83 percent of women but only 39 percent of the men identify themselves as the primary meal preparer in their households, it is not surprising that we do not observe the same relationship for the men. It would be interesting to investigate whether more time spent preparing meals by women translate into lower BMIs for other members of their households as well. Unfortunately, this question cannot be addressed with the ATUS data as only one member of each household in the sample provides time diary and BMI information.

Taken together, our findings regarding primary eating time, secondary drinking time, and time spent in food preparation and clean-up (by women) reinforce nutritional educators' emphasis on preparing meals and setting aside time where eating is one's primary focus. The role of secondary eating in healthy eating behaviours remains an open question, however.

While we did not find support for a link between physical activity and BMI, we found strong support for a link between physical *inactivity *- as measured by television/video viewing time - and BMI. This finding is consistent with past research [8-10] and with public health programs that encourage individuals to spend less time watching television/videos and more time being physically active [50].

While our 24-hour diary may be too short to capture typical time spent being physically active each day, this is not true for television/video viewing time which is sufficiently prevalent to be adequately measured with a single, 24-hour diary. Indeed, it may be that television/viewing time is a more general marker for a sedentary lifestyle that could be used in place of the more infrequent physical activity time when analyzing 24-hour time diary data.

Our reduced form model estimates provide some insights regarding the role that changing prices, opportunity costs, and nonwage income may be playing in the rising overweight/obesity epidemic. Clearly, these economic factors matter. In the case of opportunity costs, we show that an increase in the hourly opportunity cost of time is associated with a significantly lower BMI for both women and men. It suggests that the recent economic recession, which precipitated a decline in workers' opportunity costs, may lead to more weight gain for Americans. And, this may be especially true for newly unemployed individuals who are drawing down on their savings that historically was a source of interest (i.e., nonwage) income. Indeed, it would appear that rising wage rates are not just good for the economy. They may also be good for Americans' weight management.

Finally, grocery prices are inversely related to BMI for both males and females. This is consistent with past research that has linked the historical drop in prices for energy-rich, processed foods to rising BMI in the United States [51,52]. It also suggests a dilemma for policy makers. Lower food prices may increase food access, but at the same time they may also be serving to fuel greater caloric intake.

Our study results must be tempered with a couple of caveats. First, our proxies for biological differences in BMI are often statistically significant and there are clear sex-specific interactions with time use that merit further exploration. Although sample size limitations prevent us from exploring age and race/ethnicity time-use interactions, such research could provide valuable insights about the correlates of healthy body weight.

Second, our analysis presents a cautionary tale regarding the use of "small window" measures of physical activity time as it relates to BMI. Only about one-third of the women and two-fifths of the men in our ATUS sample report doing any 10-minute spells of physical activity that generate 3.3 METs or more during the 24-hour time period (See Table 2). Recall that we do not find evidence of an inverse relationship between time spent in physical activity and BMI. This is counter to a number of past studies [4-6,15] but not surprising given that our estimates of physical activity time are likely biased toward zero. The Centers for Disease Control recommends that adults age 18-64 spend 150 minutes *per week *engaged in moderate intensity aerobic activity, or that they spend 75 minutes *per week *in vigorous aerobic activity [40]. Thus, even those who do follow these recommendations might not have been exercising on the randomly chosen diary day. Although it would be costly, future time-diary data gathering efforts should consider expanding the number of time diaries gathered for each respondent and/or asking additional questions about the usual time the respondent spends each week in certain infrequent, but potentially important activities.

## Conclusions

In this large, nationally representative data set, our analyses reveal that time use and BMI are endogenous. Cross-sectional analyses that do not adjust for endogeneity will likely misstate the true relationship between time use and BMI. Based on our instrumental variables and reduced form estimates, we conclude that Americans' time use decisions have important implications for their BMIs. The analyses suggest that both eating and beverage drinking time and context matters. In the case of women only, time spent in food preparation is inversely related to BMI while for men only, time spent sleeping is inversely related to BMI. For both men and women, sedentary time, as measured by television/video viewing time also matters. In addition, the reduced form models suggest that shifts in grocery prices, opportunity costs, and non-wage income may be contributing to alterations in time use patterns and food choices that have implications for BMI. These insights should help scholars and practitioners working in the area of weight management to better target intervention efforts.

## List of Abbreviations

BMI: body mass index; ATUS: American Time Use Survey; NHANES: National Health and Nutrition Examination Survey; METs: metabolic equivalents; OLS: ordinary least squares.

## Competing interests

The author declares that they have no competing interests.

## Authors' contributions

CDZ conceived the idea and wrote the first draft of the manuscript. RBS and CDZ analyzed the data. CDZ, RBS, and WKB all contributed to the development of the empirical approach, the analysis, and the interpretation of the results. All authors have read and approved the final manuscript.

## Endnotes

^a ^Overweight and obesity are terms used to classify individuals whose weights are greater than what health officials deem to be healthy for a given height.

^b ^There are some indications that this upward trend may be tapering off as a recent analysis of obesity trends from 1999 to 2008 found no evidence of increases during this most recent 10-year period [2].

^c ^Bertrand and Schanzenbach [19] do not include a table that shows their multivariate analyses of how secondary eating time relates to BMI. Thus, we cannot ascertain if they control for physical activity or sedentary behaviors in their analyses.

^d ^This variable includes both primary time spent eating/drinking alone and with others as preliminary investigation revealed no difference in the coefficient estimates when these time use categories were separated, and thus, we collapse them.

^e ^These latter two activities are identified as generating 3.01 and 3.26 METs respectively.

^f ^Independent variables included in the wage estimation are age, age-squared, education, northeast, northcentral, and southern regions, and rural residence. The inclusion of the random error term in the predicted values reduces the potential for multicollinearity in the subsequent analyses and collinearity diagnostics confirmed that there were no collinearity issues. The estimating equations are available from the authors upon request.

^g ^Independent variables included in the nonwage income estimation are age, age-squared, education, number of children less than age 18, African American, single female headed household, single male headed household, and southern region of residence. The inclusion of the random error term in the predicted values reduces the potential for multicollinearity in the subsequent analyses and collinearity diagnostics confirmed that there were no collinearity issues. The estimating equations are available from the authors upon request.

^h ^C2ER's cost of living index was formally called the American Chamber of Commerce Research Association's Cost of Living Index. Indeed, it is still listed as ACCRA's Cost of Living Index on the C2ER web page. There are 35 items in the ACCRA grocery price index. All but 5 of these items are foods or drinks. The 5 non-food items are boys' jeans, Lipitor, veterinary services, laundry detergent, and facial tissues. Our ACCRA data set did not contain sufficient detail to delete these five items from our overall grocery price index measure. Thus, our grocery price variable contains some measurement error. Metro areas in this data set consist of urbanized areas with 50,000 or more residents. Micro areas are communities with at least 10,000 but less than 50,000 in population.

^i ^Paid work, watching television, and socializing and communicating with others were the most common primary activities that were done while eating was a secondary activity.
